# Efficacy of chemotherapy plus immune checkpoint inhibitors in patients with non-small cell lung cancer who have rare oncogenic driver mutations: a retrospective analysis

**DOI:** 10.1186/s12885-024-12554-6

**Published:** 2024-07-15

**Authors:** Teppei Yamaguchi, Junichi Shimizu, Reiko Matsuzawa, Naohiro Watanabe, Yoshitsugu Horio, Yutaka Fujiwara

**Affiliations:** Department of Thoracic Oncology, Aichi Cancer Center Hospital, 1-1, Kanokoden, Chikusa-ku, Nagoya, Aichi 464-8681 Japan

**Keywords:** Non-small cell lung cancer, Immune checkpoint inhibitors, Immunotherapy, Driver mutation

## Abstract

**Background:**

Targeted therapy is now the standard of care in driver–oncogene-positive non-small cell lung cancer (NSCLC). Its initial clinical effects are remarkable. However, almost all patients experience treatment resistance to targeted therapy. Hence, chemotherapy is considered a subsequent treatment option. In patients with driver–oncogene-negative NSCLC, combined immune checkpoint inhibitors (ICIs) and chemotherapy as the first-line therapy has been found to be beneficial. However, the efficacy of ICI plus chemotherapy against driver–oncogene-positive NSCLC other than epidermal growth factor receptor mutation and anaplastic lymphoma kinase fusion is unclear.

**Methods:**

Using the hospital medical records, we retrospectively reviewed advanced or recurrent NSCLC patients who were treated with chemotherapy with or without ICIs at Aichi Cancer Center Hospital between January 2014 and January 2023. Patients with druggable rare mutations such as KRAS-G12C, MET exon 14 skipping, HER2 20 insertion, BRAF-V600E mutations, and ROS1 and RET rearrangements were analyzed.

**Results:**

In total, 61 patients were included in this analysis. ICI plus chemotherapy was administered in 36 patients (the ICI-chemo group) and chemotherapy in 25 patients (the chemo group). The median progression-free survival (PFS) rates were 14.0 months in the ICI-chemo group and 4.8 months in the chemo group (hazard ratio [HR] = 0.54, 95% confidence interval [CI] = 0.28–1.01). The median overall survival rates were 31.3 and 21.7 months in the ICI-chemo and chemo groups, respectively (HR = 0.70, 95% CI = 0.33–1.50). Multivariate Cox regression analysis of PFS revealed that HER2 exon 20 insertion mutation was significantly associated with a poorer PFS (HR: 2.39, 95% CI: 1.19–4.77, *P* = 0.014). Further, ICI-chemo treatment was significantly associated with a better PFS (HR: 0.48, 95% CI: 0.25–0.91, *P* = 0.025).

**Conclusion:**

ICI plus chemotherapy improves treatment efficacy in rare driver–oncogene-positive NSCLC.

**Supplementary Information:**

The online version contains supplementary material available at 10.1186/s12885-024-12554-6.

## Background

The standard of care for patients with advanced-stage non-small cell lung cancer (NSCLC) has changed significantly over the last decade. Treatment now focuses on targeted therapies and immune checkpoint inhibitors (ICIs), including anti-programmed cell death protein 1 (PD-1) and anti-programmed cell death-ligand 1 (PD-L1) antibodies [1–4]. To determine the appropriate treatment strategy, prior sequencing of oncogenic driver mutations for NSCLC, including epidermal growth factor receptor (EGFR), anaplastic lymphoma kinase (ALK), Kirsten rat sarcoma viral oncogene homolog (KRAS), c-ros oncogene 1 (ROS1), mesenchymal–epithelial transition (MET), human epidermal growth factor receptor 2 (HER2), v-raf murine sarcoma viral oncogene homolog B (BRAF), ret proto-oncogene (RET), and neurotrophic tyrosine receptor kinase, is strongly recommended [5]. Despite the initial clinical efficacy of targeted therapies, almost all patients develop treatment resistance. In patients with disease progression, cytotoxic chemotherapy, including platinum combination therapy, is recommended [6]. However, it has a relatively modest survival benefit. The median progression-free survival (PFS) of platinum plus pemetrexed is 4.2–6.4 months in patients with NSCLC harboring EGFR mutation after tyrosine kinase inhibitor failure [7]. The efficacy of ICI alone is also limited. Moreover, anti-PD-1 and anti-PD-L1 antibody monotherapy is not beneficial in terms of PFS or overall survival (OS) in patients with EGFR mutation- or ALK fusion-positive NSCLC [8–10].

ICI plus chemotherapy is now the frontline therapy in patients with driver–oncogene-negative NSCLC. Several prospective studies have reported the use of frontline ICIs and chemotherapy for EGFR/ALK-positive NSCLC. A subset analysis of the IMpower150 trial showed that ICI plus chemotherapy can be a favorable treatment option for EGFR/ALK-positive NSCLC [8]. Recent phase 3 trials, such as CheckMate 722 and KEYNOTE-789, have revealed that the clinical efficacy of ICIs plus chemotherapy is not significant in patients with EGFR mutation-positive nonsquamous NSCLC [9, 10]. Discrepancies observed between the outcomes of the KEYNOTE-789 and CheckMate 722 trials and the more favorable results noted in the IMpower 150 trial indicate that supplementing immunotherapy with vascular endothelial growth factor (VEGF) targeted therapy can augment the efficacy of EGFR mutation-positive NSCLC. However, it is important to acknowledge that the data from the IMpower 150 trial stems from a subset analysis involving a limited number of EGFR/ALK-positive cases. In contrast, the inclusion of driver–oncogene-positive patients other than EGFR mutation and ALK fusion was commonly not specified in the criteria of immunotherapy trials. Several clinical trials on ICIs plus chemotherapy should have included patients with rare mutations other than EGFR and ALK mutations. However, details about driver–oncogene mutation status were commonly unknown. Therefore, the clinical efficacy of adding ICI to chemotherapy in these patients remains unclear. To the best of our knowledge, there are no prospective studies evaluating the efficacy of ICIs plus chemotherapy in driver–oncogene-positive NSCLC other than EGFR mutation and ALK fusion.

This retrospective observational study aimed to evaluate the clinical efficacy of ICI plus platinum combination chemotherapy in patients with NSCLC harboring rare oncogenic driver mutations other than EGFR mutation and ALK fusion.

## Methods

### Patients

The medical records of patients with advanced-stage or recurrent (post-resection or post-chemoradiotherapy) NSCLC at Aichi Cancer Center Hospital between January 2014 and January 2023 were reviewed. The inclusion criteria were as follows: (1) patients pathologically diagnosed with NSCLC; (2) those with druggable rare mutations (based on either direct sequencing or next generation sequencing on validated platforms) such as KRAS-G12C, MET exon 14 skipping mutation (MET ex14), HER2 exon 20 insertion (HER2 ex20-ins), BRAF-V600E mutations, and ROS1 and RET rearrangements; (3) those who received platinum combination chemotherapy with or without ICIs as the initial cytotoxic chemotherapy for advanced-stage or recurrent NSCLC. The exclusion criteria were as follows: (1) patients with an Eastern Cooperative Oncology Group Performance Status (ECOG-PS) score of ≥ 3; (2) those with any druggable rare mutations such as resistance mutation after targeted therapy; (3) those with a previous history of ICI treatment prior to platinum combination chemotherapy (perioperative ICI treatment and durvalumab after chemoradiotherapy is acceptable). Data on clinicopathologic features and treatment history were collected from the medical records. Tumor response was assessed based on the Response Evaluation Criteria in Solid Tumors Version 1.1 [11]. The expression of PD-L1 in tumors was evaluated using pretreatment tumor biopsy specimens with the immunohistochemistry assay with PD*-*L1 22C3 or 28 − 8 PharmDx (Dako, Santa Clara, CA). PD-L1 positivity was defined as expression levels of ≥ 1% in a tumor section.

### Statistical analysis

The Fisher’s exact test was used to compare categorical variables, and the Mann–Whitney U test was used to compare continuous variables between the two groups. *P*-values for multiple comparisons were corrected using the Bonferroni method [12]. The primary endpoint was PFS, defined as the interval between the first day of first-line treatment with platinum combination chemotherapy with or without ICI and the day of clinical or radiographic disease progression or death. The secondary endpoint was OS, defined the interval between the first day of platinum combination chemotherapy with or without ICI and the day of death from any cause. Survival analysis was performed using the Kaplan–Meier method and data were compared via the log-rank test. Univariate and multivariate analyses using the Cox proportional hazard model were applied to calculate the hazard ratio (HR) and its 95% confidence interval (CI). Parameters with a *P* value of < 0.1 in the univariate analysis were included in the multivariate analysis. In an exploratory analysis limited to patients with known PD-L1 levels, PD-L1 levels were included in the multivariate analysis. All analyses were performed using EZR ver1.54 (Saitama Medical Center, Jichi Medical University, Saitama, Japan), which is an open-source software program based on R (The R Foundation for Statistical Computing, Vienna, Austria) [13]. The analysis cutoff date was May 15, 2023.

## Results

### Characteristics of the patients

**Fig. 1:**
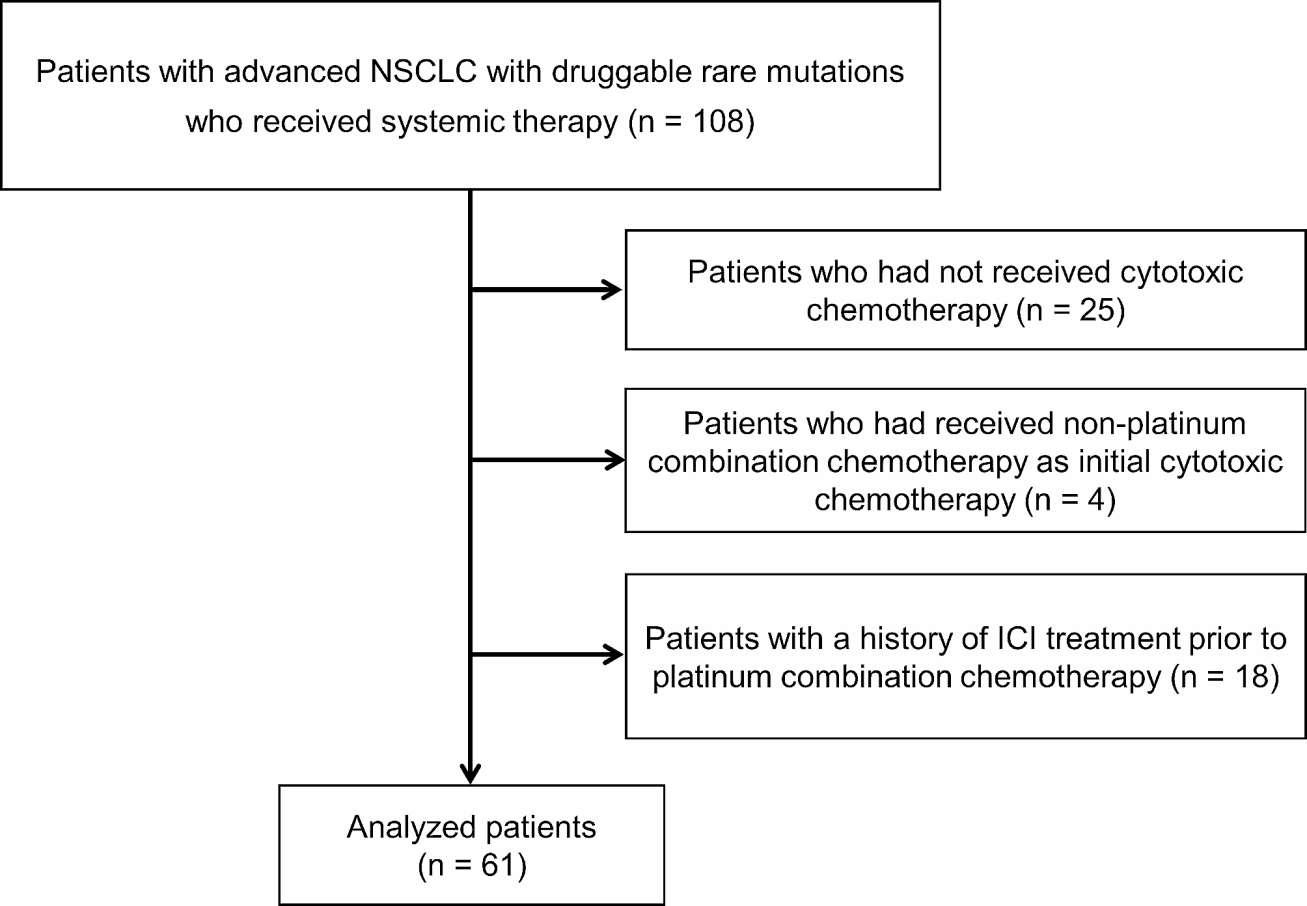
Patient flowchart NSCLC: non-small cell lung cancer; ICI: immune checkpoint inhibitor

From 108 consecutive patients with advanced NSCLC with druggable rare mutations who had received systemic therapy, 25 who had not received cytotoxic chemotherapy, four who had received non-platinum combination chemotherapy as initial cytotoxic chemotherapy, and 18 with a history of ICI treatment prior to platinum combination chemotherapy were excluded; consequently, the final study cohort comprised 61 patients (Fig. 1). Overall, 36 were treated with ICI plus platinum combination chemotherapy (ICI-chemo group), and 25 patients received platinum combination chemotherapy (chemo group). Table 1 and Supplementary Table S1 show the characteristics of the patients and treatment regimen. No significant differences were observed between patients with PD-L1 < 1% and those with PD-L1 ≥ 1%. However, when patients were categorized into three groups based on PD-L1 values, including PD-L1 < 1%, 1–49%, and ≥ 50%, significantly more patients had PD-L1 ≥ 50% compared with the number of patients with PD-L1 1–49% in the ICI-chemo group (using Fisher’s exact test and Bonferroni correction) (Supplementary Table S2). No other significant differences were detected difference between the two groups in terms of demographic features including age, sex and clinical characteristics such as smoking status, ECOG-PS score, disease stage, histology, prior treatment lines, and oncogenic mutation type. The median age of all patients was 63 (range: 25–81) years. Most patients were men (59.0%) and current/ex-smokers (59.0%) and had a ECOG-PS score of 0–1 (91.8%). Approximately 24.6% of patients had a previous history of targeted therapy, and 23.0% received initial targeted therapy after platinum combination therapy with or without ICIs. In Japan, ICI plus chemotherapy was approved for patients with unresectable advanced or recurrent NSCLC in December of 2018. All patients initially chose chemotherapy alone before the approval of ICI plus chemotherapy, but the majority of patients (87.8%) opted for ICI plus chemotherapy after its approval, regardless of PD-L1 expression (Supplementary Tables S3 and S4).

**Table 1:** Characteristics of the patients

Characteristics	All patients *n* = 61 *n* (%)	Patients receiving ICI-chemo *n* = 36 *n* (%)	Patients receiving chemo *n* = 25 *n* (%)	*P*-value
Age, years				
Median (range)	63 (25–81)	64 (25–81)	63 (29–81)	0.75
< 65	32 (52.5)	19 (52.8)	13 (52.0)	1.000
≥ 65	29 (47.5)	17 (47.2)	12 (48.0)	
Sex				0.79
Male	36 (59.0)	22 (61.1)	14 (70.8)	
Female	25 (41.0)	14 (38.9)	11 (44.0)	
Smoking status				0.29
Current/ex-smoker	36 (59.0)	19 (52.8)	17 (68.0)	
Never-smoker	25 (41.0)	17 (47.2)	8 (32.0)	
Performance status score				0.072
0–1	56 (91.8)	31 (86.1)	25 (100.0)	
≥ 2	5 (8.2)	5 (13.9)	0	
Stage				0.56
IIIB–IV	41 (67.2)	25 (69.4)	16 (64.0)	
Recurrence after surgery	17 (27.9)	10 (27.8)	7 (28.0)	
Histology				0.29
Adenocarcinoma	51 (83.6)	24 (96.0)	27 (75.0)	
Squamous cell carcinoma	1 (1.6)	0 (0.0)	1 (2.8)	
Pleomorphic carcinoma	3 (4.9)	0 (0.0)	3 (8.3)	
LCNEC	1 (1.6)	0 (0.0)	1 (2.8)	
Not otherwise specified	5 (8.2)	1 (4.0)	4 (11.1)	
Recurrence after CRT	3 (4.9)	1 (2.8)	2 (8.0)	
With previous target therapy				0.24
Yes	15 (24.6)	11 (30.6)	4 (16.0)	
No	46 (75.4)	25 (69.4)	21 (84.0)	
Received initial targeted therapy after platinum combination therapy				0.54
Yes	14 (23.0)	7 (19.4)	7 (28.0)	
No	47 (77.0)	29 (80.6)	18 (72.0)	
Oncogenic mutation type				0.98
ROS1	11 (18.0)	6 (16.7)	5 (20.0)	
BRAF-V600E	5 (8.2)	3 (8.3)	2 (8.0)	
HER2 exon 20 insertion	15 (24.6)	9 (25.0)	6 (24.0)	
KRAS-G12C	18 (29.5)	10 (27.8)	8 (32.0)	
MET exon 14 skipping	10 (16.4)	7 (19.4)	3 (12.0)	
RET	2 (3.3)	1 (2.8)	1 (4.0)	
PD-L1 expression levels				0.46*
< 1%	10 (16.4)	6 (16.7)	4 (16.0)	
≥ 1%	40 (65.6)	29 (80.6)	11 (44.0)	
1–49%	16 (26.2)	8 (22.2)	8 (32.0)	
≥ 50%	24 (39.3)	21 (58.3)	3 (12.0)	
(Unknown)	11 (18.0)	1 (2.8)	10 (40.0)	

* Analysis was performed after excluding cases in which PD-L1 expression was unknown

ICI: immune checkpoint inhibitor; chemo: chemotherapy; CRT: chemoradiotherapy; LCNE: large cell neuroendocrine carcinoma; PD-L1: programmed cell death-ligand 1

### Clinical outcomes

**Fig. 2:**
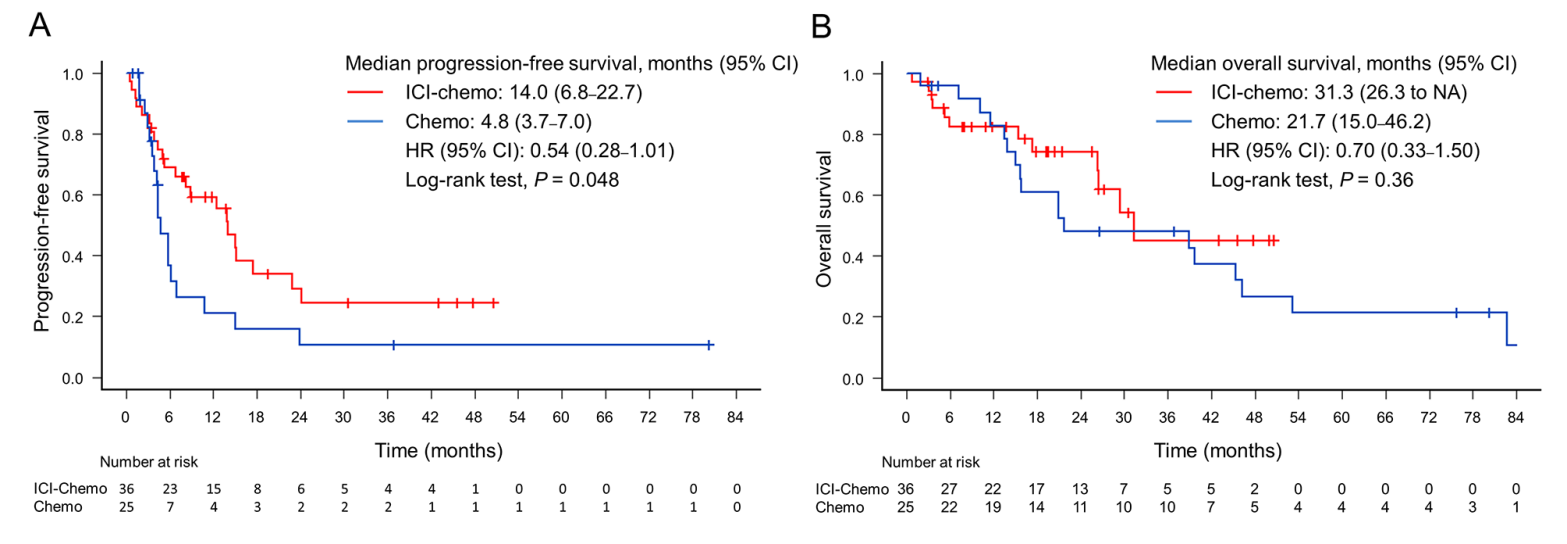
Kaplan–Meier survival curves of progression-free survival and overall survival in all patients (**A** and **B**) Chemo: chemotherapy; ICI: immune checkpoint inhibitor; CI: confidence interval

**Fig. 3:**
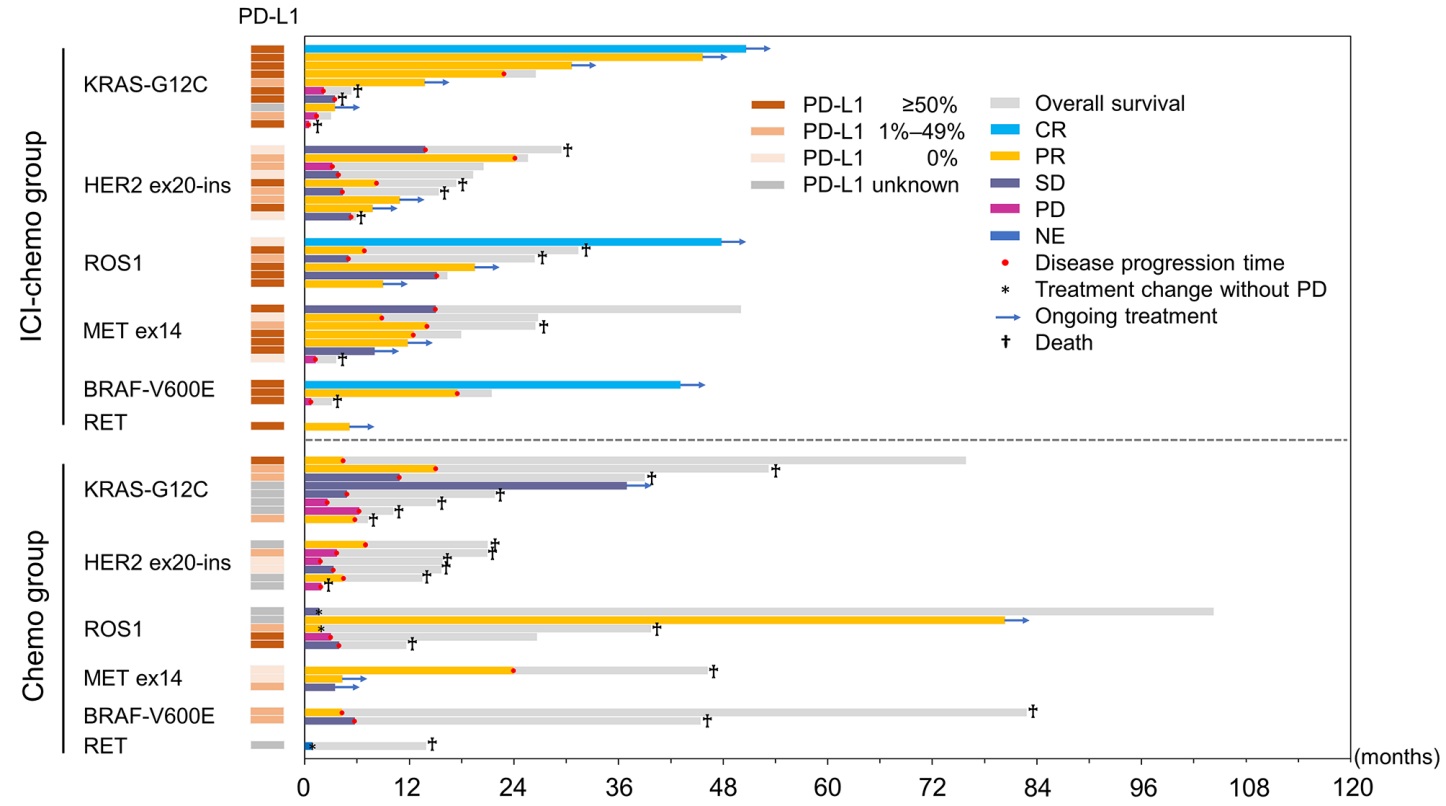
Swimmer plots of 61 patients who received chemotherapy with or without immune checkpoint inhibitors Individual swimmer plots displayed the mutation types and treatment duration. Chemo: chemotherapy; ICI: immune checkpoint inhibitor; ex20-ins: exon 20 insertion; ex14: exon 14 skipping; CR: complete response; PR: partial response; SD: stable disease; PD: progressive disease

The median PFS were 14.0 months in the ICI-chemo group and 4.8 months in the chemo group (HR = 0.54; 95% CI = 0.28–1.01, *P* = 0.048, log-rank test, *P* = 0.052, Cox-regression model) (Fig. 2A; Table 2). The median OS were 31.3 months in the ICI-chemo group and 21.7 months in the chemo group (HR = 0.70; 95% CI = 0.33–1.50, *P* = 0.36, log-rank test) (Fig. 2B). The overall response rate (ORR) of the ICI-chemo group was 61.1% (95% CI = 43.5–76.9). This included 8.3% of patients with complete response, 52.8% with partial response, 22.2% with stable disease, and 16.7% with progressive disease. The ORR of the chemo group was 40.0% (95% CI = 21.1–61.3). This included 40.0% of patients with partial response, 32.0% with stable disease, 24.0% with progressive disease, and 4% who were not evaluated (*P* = 0.12). Figure 3 shows the swimmer plots of 61 patients who received platinum combination chemotherapy with or without ICIs for advanced-stage or recurrent disease. The median PFS rates of patients with KRAS-G12C, HER2 ex20-ins, ROS1, MET ex14, and BRAF-V600E were 22.9, 8.2, 15.2, 12.5, and 17.5 months in the ICI-chemo group, and 6.0, 3.5, 3.9, 23.9, and 5.0 months in the chemo group, respectively. Only one patient each in the ICI-chemo and chemo groups presented with RET. One patient in the ICI-chemo group had ongoing treatment at a median of 5.2 months, and the PFS of patient in the chemo group was 0.9 months.

**Table 2:** Univariate and multivariate Cox regression analyses of progression-free survival after chemotherapy with or without immune checkpoint inhibitors

		Univariate model			Multivariate model		
		HR	(95% CI)	*P*-value	HR	(95% CI)	*P*-value
Age, years	≥ 65 vs. <65	1.06	(0.57–1.98)	0.84			
Sex	Female vs. male	0.77	(0.41–1.47)	0.43			
Smoking status	Current/ex-smoker vs. never-smoker	1.63	(0.86–3.11)	0.13			
Performance status score	≥ 2 vs. 0 or 1	1.50	(0.53–4.22)	0.45			
Treatment	ICI-chemo vs. Chemo	0.54	(0.28–1.01)	0.052	0.48	(0.25–0.91)	0.025
Bevacizumab	Use vs. not use	1.08	(0.47–2.44)	0.86			
Histology	Adenocarcinoma vs. others	0.76	(0.33–1.72)	0.51			
Oncogenic mutation	ROS1	0.57	(0.22–1.45)	0.24			
	BRAF-V600E	1.09	(0.39–3.08)	0.87			
	HER2 exon 20 insertion	2.12	(1.08–4.16)	0.030	2.39	(1.19–4.77)	0.014
	KRAS-G12C	0.87	(0.44–1.73)	0.70			
	MET exon 14 skipping	0.82	(0.34–1.96)	0.65			
Stage	Recurrence vs. stage IIIB–IV	1.14	(0.59–2.19)	0.69			

Chemo: chemotherapy; CI: confidence interval; HR: hazard ratio; ICI: immune checkpoint inhibitor

Univariate and multivariate Cox regression analyses of PFS were conducted considering factors such as age, sex, smoking status, ECOG-PS score, stage, treatment (ICI-chemo vs. chemo), use of bevacizumab, and type of oncogenic mutations (Table 2). In the multivariate analysis, HER2 ex20-ins mutation was significantly related to poor PFS (HR: 2.39, 95% CI: 1.19–4.77, *P* = 0.014). ICI-chemo treatment was significantly correlated with a better PFS (HR: 0.48, 95% CI: 0.25–0.91, *P* = 0.025).

**Fig. 4:**
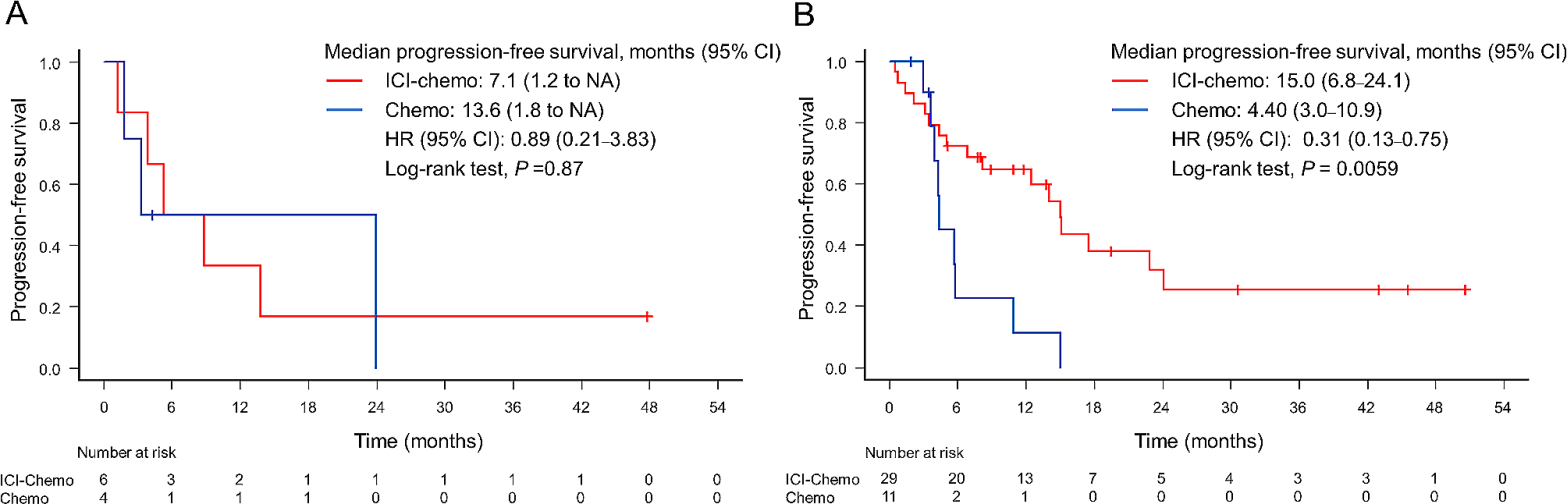
Progression-free survival in patients who received platinum combination chemotherapy with or without immune checkpoint inhibitors (**A**) PD-L1 of < 1% and (**B**) PD-L1 of ≥ 1%. Chemo: chemotherapy; ICI: immune checkpoint inhibitor; CI: confidence interval; PD-L1: programmed cell death-ligand 1

An exploratory analysis was performed only in patients with PD-L1 status. In total, 35 patients in the ICI-chemo group and 15 in the chemo group presented with PD-L1 status (Fig. 4A and B). Using a cutoff value of 1%, 29 of 35 patients in the ICI-chemo group and 11 of 15 patients in the chemo group were positive for PD-L1. In PD-L1-negative patients, the PFS curve of the ICI-chemo group did not significantly differ from that of the chemo group (HR: 0.89, 95% CI: 0.21–3.83, *P* = 0.87, log-rank test). PD-L1-positive patients in the ICI-chemo group had a significantly better PFS than those in the chemo group (HR: 0.31, 95% CI: 0.13–0.75, *P* = 0.0059, log-rank test). Univariate and multivariate Cox regression analyses were conducted to assess PFS in patients with documented PD-L1 levels as part of an exploratory analysis. The results closely resembled those of the primary analysis, indicating that ICI-chemo was a factor significantly associated with prolonged PFS, whereas HER2 ex20-ins emerged as a factor significantly associated with shortening of PFS (Supplementary Table S5).

## Discussion

This study revealed that ICI plus chemotherapy prolonged PFS in patients with NSCLC who presented with rare oncogenic driver mutations other than EGFR mutation or ALK rearrangement. The IMMUNOTARGET registry study showed that the efficacy of ICIs is limited in patients with EGFR mutation or ALK rearrangement [14]. Specifically, the objective response rate of patients with ALK rearrangement was 0% (0 of 23). In the prospective randomized phase 2 WJOG8515L trial, patients with advanced EGFR-mutated NSCLC who acquired resistance to EGFR-TKIs without T790M mutation were randomized to receive either nivolumab or carboplatin-pemetrexed. Results showed that the therapeutic effect of nivolumab was inferior to that of platinum combination chemotherapy [15]. In the phase 2 ATLANTIC trial that investigated the efficacy of the anti-PD-L1 antibody durvalumab in advanced-stage NSCLC, the response rate was 3.6% in patients with a PD-L1 expression of < 25% and 12.2% in those with a PD-L1 expression of > 25% and those with EGFR or ALK mutations [16]. Particularly, the response rate of patients with ALK fusion was 0% (0 of 10). Therefore, patients with EGFR mutation or ALK rearrangement were excluded from prospective clinical trials of ICIs because of the expected low efficacy. However, several prospective clinical trials have recently reported that ICI plus chemotherapy was effective against EGFR mutation-positive NSCLC. In an exploratory subgroup analysis in the phase 3 IMpower150 study, the VEGF inhibitor bevacizumab and the PD-L1 inhibitor atezolizumab combined with chemotherapy were clinically effective in patients with tyrosine kinase inhibitor-treated advanced-stage EGFR mutation- or ALK rearrangement-positive NSCLC [8, 17]. Recently, ORIENT-31, a prospective, double-blind, and multicenter phase 3 trial, revealed that combined anti-PD-1 antibody and anti-VEGF antibody treatments with chemotherapy significantly prolonged PFS compared with chemotherapy alone in patients with EGFR-mutant nonsquamous NSCLC who had disease progression after EGFR-TKI therapy [18]. In contrast, the multicenter, prospective phase 3 clinical trials CheckMate 722 and KEYNOTE-789, which aimed to validate the efficacy of ICI plus chemotherapy in patients with NSCLC who presented with EGFR mutation, showed a trend of prolonged PFS. However, significant differences were not detected [18]. The difference between ORIENT-31 and Checkmate 722/KEYNOTE-789 was the addition of the VEGF inhibitor. Hence, the significantly prolonged PFS in the ORIENT-31 trial was caused by the combined use of ICI and VEGF inhibitors.

There are limited reports on the effects of ICIs in patients with NSCLC who present with rare oncogenic driver mutations other than EGFR mutation and ALK rearrangement. The outcomes of rare mutations were rarely reported in large clinical trials assessing ICIs and combined chemotherapy in unselected patient cohorts with advanced-stage NSCLC. However, several subgroup analyses of large clinical trial data were performed for patients with KRAS-mutated tumors. In the IMpower150 trial, a subgroup analysis of patients with KRAS mutations revealed that PFS and OS were longer in the atezolizumab plus chemotherapy group than in the chemotherapy alone group [19]. Similarly, in the KEYNOTE-189 trial, a post-hoc analysis revealed that patients with NSCLC who presented with any KRAS mutations and those with the KRAS-G12C mutation who received pembrolizumab plus chemotherapy achieved better outcomes than those who received chemotherapy alone [20]. According to our results, ICI-chemo exhibited good antitumor effects in patients with the KRAS-G12C mutation. Combination therapy with adagrasib, a KRAS-G12C inhibitor, and pembrolizumab, an anti-PD-1 antibody, resulted in favorable outcomes. Therefore, further therapeutic development is expected [21].

The efficacy of ICIs in NSCLC patients with other rare mutations has been reported in several retrospective studies [14, 22–24]. A recent retrospective lung cancer consortium study conducted at the University of California compared the clinical benefits of ICI plus chemotherapy with those of chemotherapy alone in patients with NSCLC who presented with oncogenic drivers. In contrast to our results, the aforementioned study showed no clinical benefit of ICI in terms of PFS and OS [25]. This difference may be attributed to the type of oncogenic driver mutations. In a previous study conducted by the University of California Lung Cancer Consortium, 54.9% of patients had EGFR mutations, 32.9% of patients had KRAS mutations, 5.3% of patients had ALK fusions, and < 3% of patients had other mutations (HER2, MET, RET, ROS1, or BRAF-non-V600E). Meanwhile, our study excluded patients with EGFR mutations and ALK fusions. KRAS-G12C, HER2, MET, and ROS1 mutations were predominant, and BRAF mutations only included V600E. Moreover, the PFS of the ICI-chemo group was significantly longer than that of the chemo group. Although the number of cases in each driver–oncogene subset was small, the median PFS was more likely to be longer in all mutation types except MET ex14 skipping. This result is challenging to interpret because patients had a variety of genetic mutations and fusions. BRAF-, MET-, and KRAS-G12C-positive patients were more likely to be smokers compared with EGFR/ALK-positive patients who can be managed with ICIs [26–28]. In our study, the ICI-chemo group was more likely to have higher PD-L1 expression than the chemo group, and this difference may have affected our results. Notably, PFS was significantly longer in patients with a PD-L1 of ≥ 1% in the ICI-chemo than in those in the chemo group. Therefore, ICIs plus chemotherapy can have clinical benefits in patients who are positive for PD-L1 and those with a smoking history. Although the addition of ICI to chemotherapy extended PFS, there was no significant difference in OS between the two groups. Notably, the proportion of patients in the chemo group (28.0%) who received initial targeted therapy after platinum combination therapy was slightly higher than that in the ICI-chemo group (19.4%). Both groups included patients who did not receive targeted therapy. However, most patients in the chemo group started treatment before 2018. The corresponding targeted therapy may have been approved at a later date, resulting in targeted therapy as a late-line treatment. Targeted therapy impacts the survival of patients with driver mutation-positive NSCLC. Therefore, the lack of a significant difference in OS may have been due to the late-line treatment, which overshadowed the influence of ICI.

The results of the multivariate analysis highlight HER2 mutations (ex20-ins) as a significant factor associated with shortened PFS among NSCLC cases with rare genetic mutations. Several retrospective studies have focused on the impact of platinum combination therapy in HER2-positive cases [29–31]. In HER2-mutant patients with NSCLC undergoing first-line pemetrexed-based chemotherapy, the ORR was 36% and median PFS was 5.1 months, resembling the KRAS-mutant/EGFR-mutant group in terms of PFS but differing significantly from the ALK/ROS1 rearrangement group (*p* = 0.004) [29]. In another retrospective study, patients with HER2-mutant NSCLC showed poor response to ICIs. The ORR and median PFS were 0% and 1.6 months with ICIs alone and 20% and 5.0 months with ICIs plus chemotherapy, respectively [31]. Our study results indicate the reduced efficacy of platinum combination therapy ± ICIs in HER2-positive cases. However, it is important to note that this study is based on a limited number of cases, indicating the need for further research in large-scale studies.

The current study had several limitations. Due to its retrospective design and small sample size, there was heterogeneity in chemotherapy regimens, scan intervals, and characteristics of the patients. As ICI plus chemotherapy was approved in December 2018, the decision of whether to add ICI to chemotherapy was made at the discretion of the attending physician, resulting in treatment selection bias. Moreover, the OS analysis must be interpreted with caution because many patients in the chemotherapy group had started treatment earlier, and therefore, had fewer molecular targeted drugs available than patients in the ICI plus chemotherapy group. Owing to the limited patient cohort, an analysis based on driver–oncogene mutation was not feasible. Furthermore, the inclusion of all factors in the multivariate analysis was constrained by the small sample size, and only factors showing associations in the univariate analysis were included in the multivariate analysis. There were several cases in which PD-L1 testing was not performed. Therefore, the PD-L1 expression rate could not be included in the multivariate analysis. Additionally, the proportion of patients with high PD-L1 expression was lower in the chemo group, possibly because some patients with high PD-L1 expression received pembrolizumab monotherapy. Despite these limitations, to the best of our knowledge, this is the first study to show the benefits of ICI plus chemotherapy in terms of PFS in patients with NSCLC who present with rare oncogenic driver mutations other than EGFR mutations and ALK fusion.

## Conclusions

Patients with NSCLC harboring rare driver oncogenes, excluding EGFR mutations and ALK fusion, who received ICI-chemo were more likely to have a better PFS. However, this was a retrospective study with a small number of patients. Therefore, to validate our findings, prospective trials with a larger number of patients should be performed.

### Electronic supplementary material

Below is the link to the electronic supplementary material.

Supplementary Material 1

## Data Availability

No datasets were generated or analysed during the current study.

## Abbreviations

NSCLC: non-small cell lung cancer
ICI: immune checkpoint inhibitor
EGFR: epidermal growth factor receptor
ALK: anaplastic lymphoma kinase
KRAS: Kirsten rat sarcoma viral oncogene homolog
ROS1: c-ros oncogene 1
MET: mesenchymal-epithelial transition
ex14: exon 14 skipping
HER2: human epidermal growth factor receptor 2
ex20-ins: exon 20 insertion
BRAF: v-raf murine sarcoma viral oncogene homolog B
RET: ret proto-oncogene
PFS: progression-free survival
PD-1: programmed cell death protein 1
PD-L1: programmed cell death-ligand 1
OS: overall survival
ECOG-PS: Eastern Cooperative Oncology Group Performance Status
HR: hazard ratio
CI: confidence interval
chemo: chemotherapy
ORR: overall response rate
CR: complete response
PR: partial response
SD: stable disease
PD: progressive disease
VEGF: vascular endothelial growth factor
