# Supplementary material for: Efficacy of chemotherapy plus immune checkpoint inhibitors in patients with non-small cell lung cancer who have rare oncogenic driver mutations: a retrospective analysis
Source: BMC Cancer. 2024 Jul 15;24:842. doi: 10.1186/s12885-024-12554-6 (PMC11247748; doi:10.1186/s12885-024-12554-6)
Supplement: Supplementary file 1 — Supplementary Material 1 [file 12885_2024_12554_MOESM1_ESM.docx]

**Supplemental Table S1: Details of treatment regimens**

| **Treatment** | **ICI-chemotherapy**  **n = 36 (%)** |
| --- | --- |
| Platinum + pemetrexed + pembrolizumab | 27 (75.0) |
| Carboplatin + paclitaxel/nab-paclitaxel + pembrolizumab | 2 (5.6) |
| Carboplatin + paclitaxel + bevacizumab + atezolizumab | 2 (5.6) |
| Carboplatin + nab-paclitaxel + atezolizumab | 3 (8.3) |
| Platinum + pemetrexed + ipilimumab + nivolumab | 2 (5.6) |

| **Treatment** | **Chemotherapy**  **n = 25 (%)** |
| --- | --- |
| Platinum + pemetrexed + bevacizumab | 8 (32.0) |
| Carboplatin + paclitaxel + bevacizumab | 2 (8.0) |
| Platinum + pemetrexed | 12 (48.0) |
| Carboplatin + nab-paclitaxel | 2 (8.0) |
| Carboplatin + S-1 | 1 (4.0) |

ICI, immune checkpoint inhibitor

**Supplemental Table S2. Bonferroni correction table**

|  | ***P*-value** | |
| --- | --- | --- |
|  | **PD-L1**  **< 1%** | **PD-L1**  **1%–49%** |
| PD-L1  1%–49% | 1.00 | - |
| PD-L1  ≥ 50% | 0.47 | 0.041* |
| *Statistical significance at the 5% level | | |

PD-L1, programmed cell death-ligand 1.

|  | **N** | **Pre-ICI-Chemo approval, n, (%)** | **Post-ICI-Chemo approval, n, (%)** | **P-value** |
| --- | --- | --- | --- | --- |
| ICI-Chemo | 36 | 0 | 36 (87.8) | <0.001 |
| Chemo | 25 | 20 (100.0) | 5 (12.2) |  |

**Supplemental Table S3. Treatment selection before and after ICI-chemo approval**

Chemo, chemotherapy; ICI, immune checkpoint inhibitor.

**Supplemental Table S4. Treatment selection based on PD-L1 expression before and after ICI-chemo approval**

|  | **n** | **PD-L1**  **<1%,**  **n, (%)** | **PD-L1**  **1%–49%, n, (%)** | **PD-L1**  **≥50%,**  **n, (%)** | **PD-L1**  **unknown,** **n, (%)** |
| --- | --- | --- | --- | --- | --- |
| **Pre-ICI-Chemo approval** |  |  |  |  |  |
| ICI-Chemo | 0 | 0 | 0 | 0 | 0 |
| Chemo | 20 | 2(100.0) | 7 (100.0) | 2 (100.0) | 9(100.0) |
| **Post-ICI-Chemo approval** |  |  |  |  |  |
| ICI-Chemo | 36 | 6 (75.0) | 8 (88.9) | 21 (95.5) | 1 (50.0) |
| Chemo | 5 | 2 (25.0) | 1 (11.1) | 1 (4.5) | 1 (50.0) |

Chemo, chemotherapy; ICI, immune checkpoint inhibitor; PD-L1, programmed cell death-ligand 1.

| **Supplemental Table S5. Univariate and multivariate Cox regression analyses of progression-free survival after chemotherapy with or without immune checkpoint inhibitors** **in patients whose PD-L1 expression was measured** | | | | | | | |
| --- | --- | --- | --- | --- | --- | --- | --- |
|  |  | **Univariate model** | | | **Multivariate model** | | |
|  |  | **HR** | **(95% CI)** | ***P*-value** | **HR** | **(95% CI)** | ***P*-value** |
| **Age, years** | ≥65 vs. <65 | 1.08 | (0.55–2.12) | 0.82 |  |  |  |
| **Sex** | Female vs. male | 0.74 | (0.37–1.48) | 0.39 |  |  |  |
| **Smoking status** | Current/ex-smoker vs. never-smoker | 1.80 | (0.89–3.65) | 0.103 |  |  |  |
| **Performance status score** | ≥2 vs. 0 or 1 | 1.48 | (0.52–4.21) | 0.47 |  |  |  |
| **Treatment** | ICI-chemo vs. Chemo | 0.43 | (0.21–0.89) | 0.023 | 0.36 | (0.16–0.78) | 0.010 |
| **Histology** | Adenocarcinoma vs. others | 0.82 | (0.33–1.99) | 0.66 |  |  |  |
| **Bevacizumab** | Use vs. not use | 1.51 | (0.62–3.66) | 0.37 |  |  |  |
| **Oncogenic mutation type** | ROS1 | 0.75 | (0.29–1.95) | 0.56 |  |  |  |
|  | BRAF-V600E | 1.06 | (0.37–3.01) | 0.92 |  |  |  |
|  | HER2 exon 20 insertion | 1.88 | (0.89–3.98) | 0.099 | 2.38 | (1.07–5.29) | 0.034 |
|  | KRAS-G12C | 0.82 | (0.38–1.76) | 0.60 |  |  |  |
|  | MET exon 14 skipping | 0.85 | (0.35–2.08) | 0.73 |  |  |  |
| **PD-L1 expression** | Positive vs. negative | 0.72 | (0.32–1.59) | 0.41 | 1.01 | (0.44–2.31) | 0.99 |
| **Stage** | Recurrence vs. stage IIIB–IV | 1.50 | (0.75–2.98) | 0.25 |  |  |  |
| Chemo, chemotherapy; CI, confidence interval; HR, hazard ratio; ICI, immune checkpoint inhibitor; PD-L1, programmed cell death-ligand 1. | | | | | | | |
